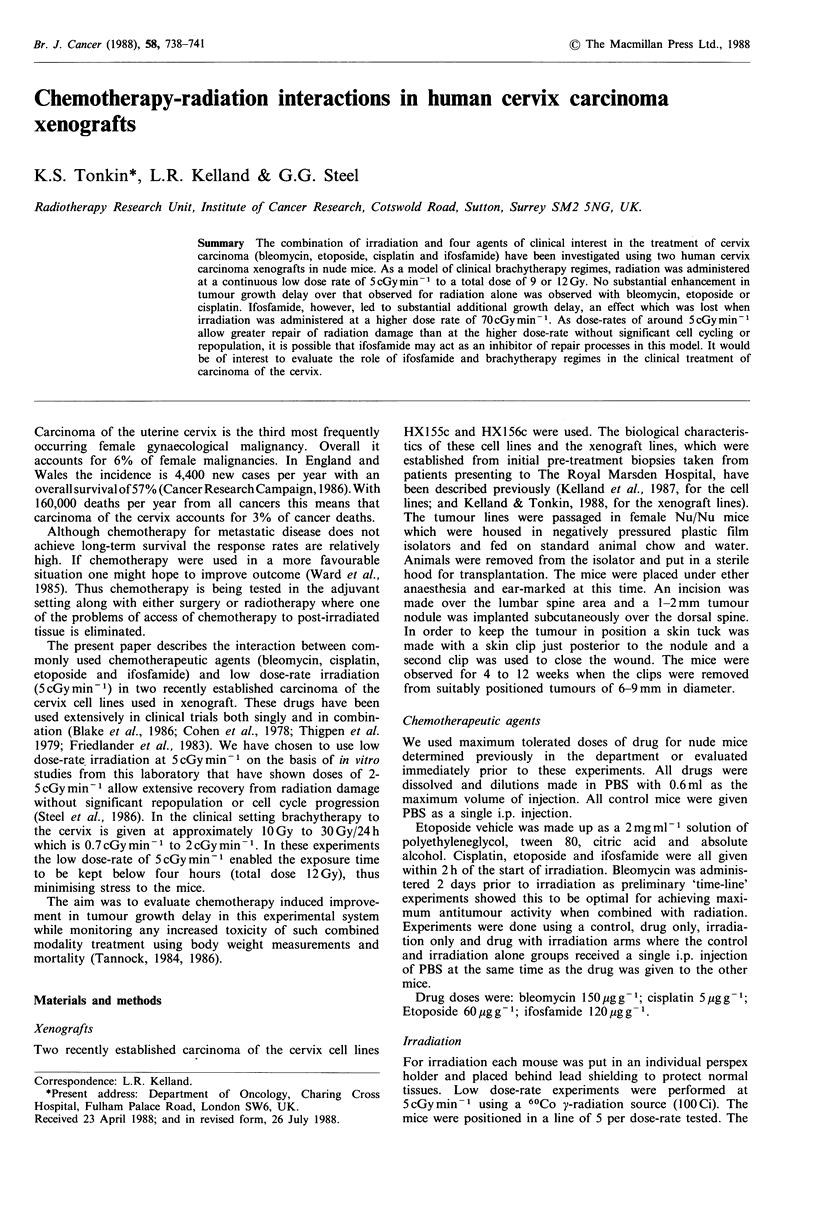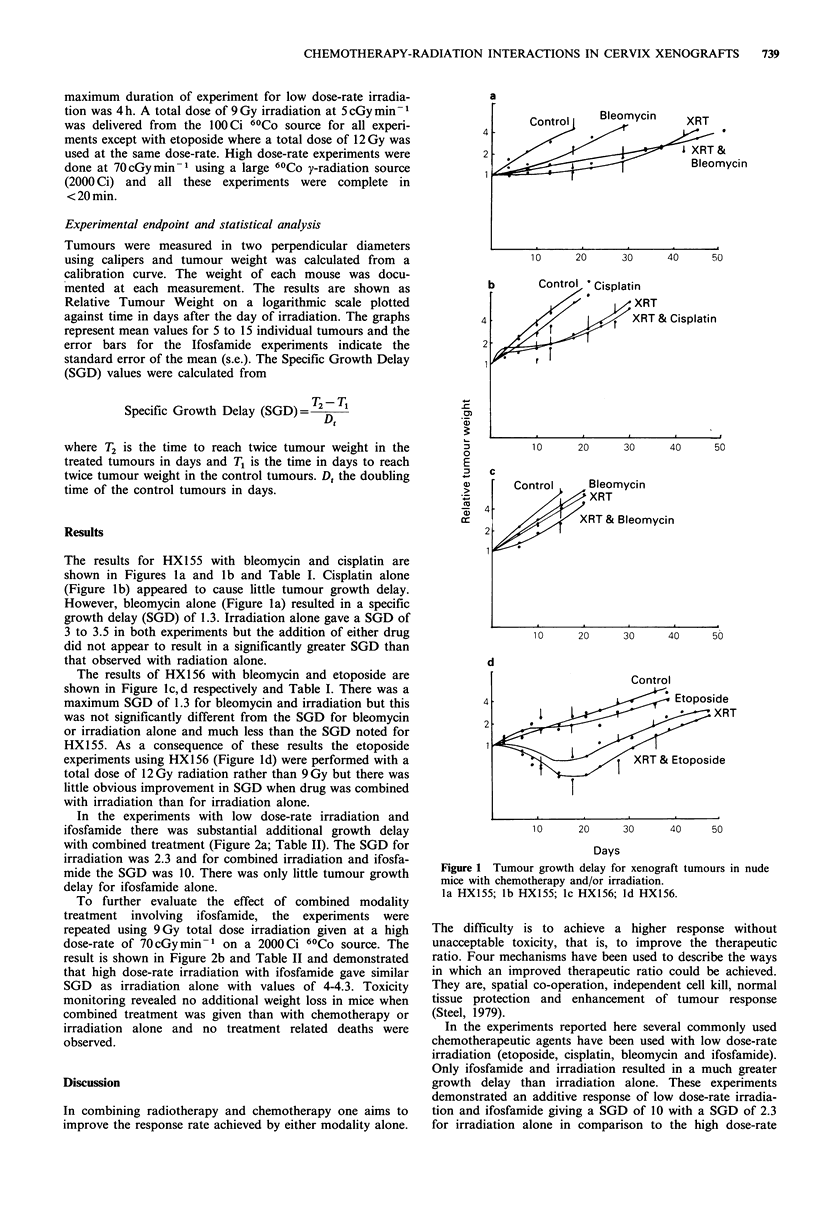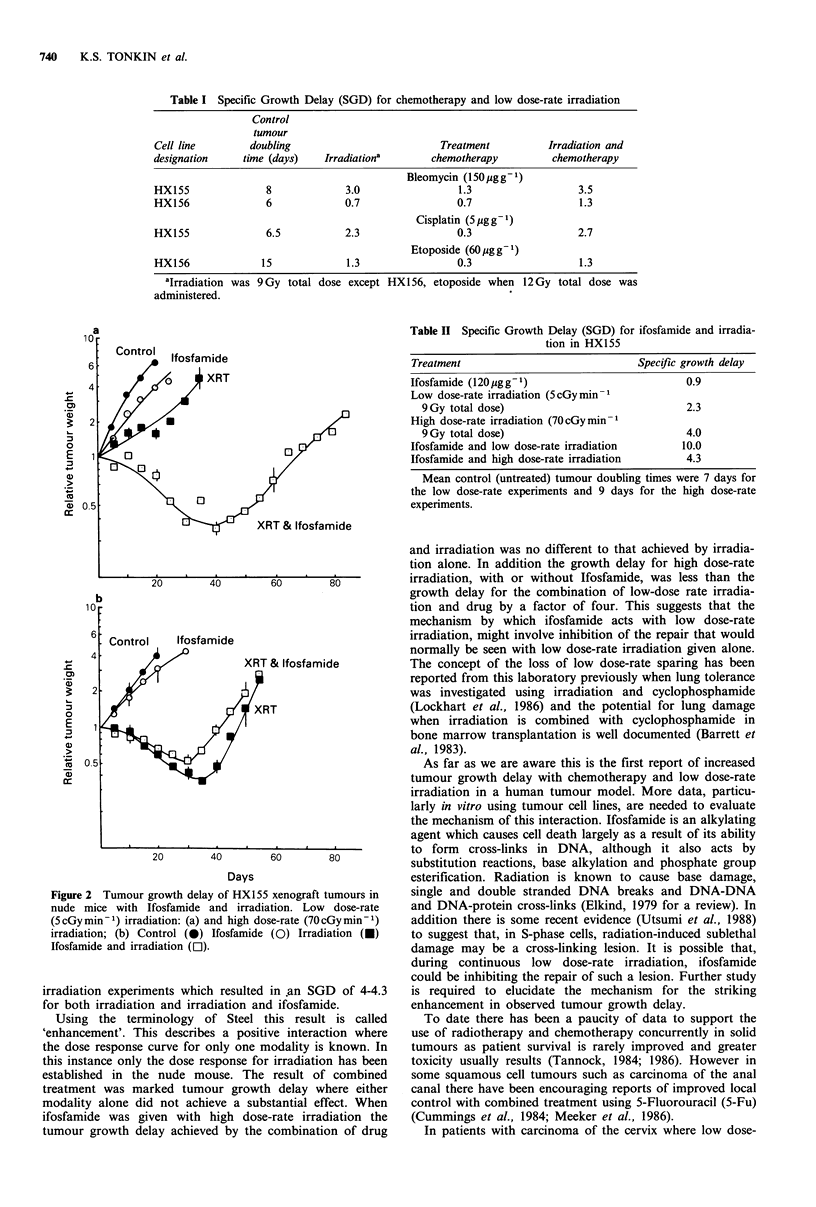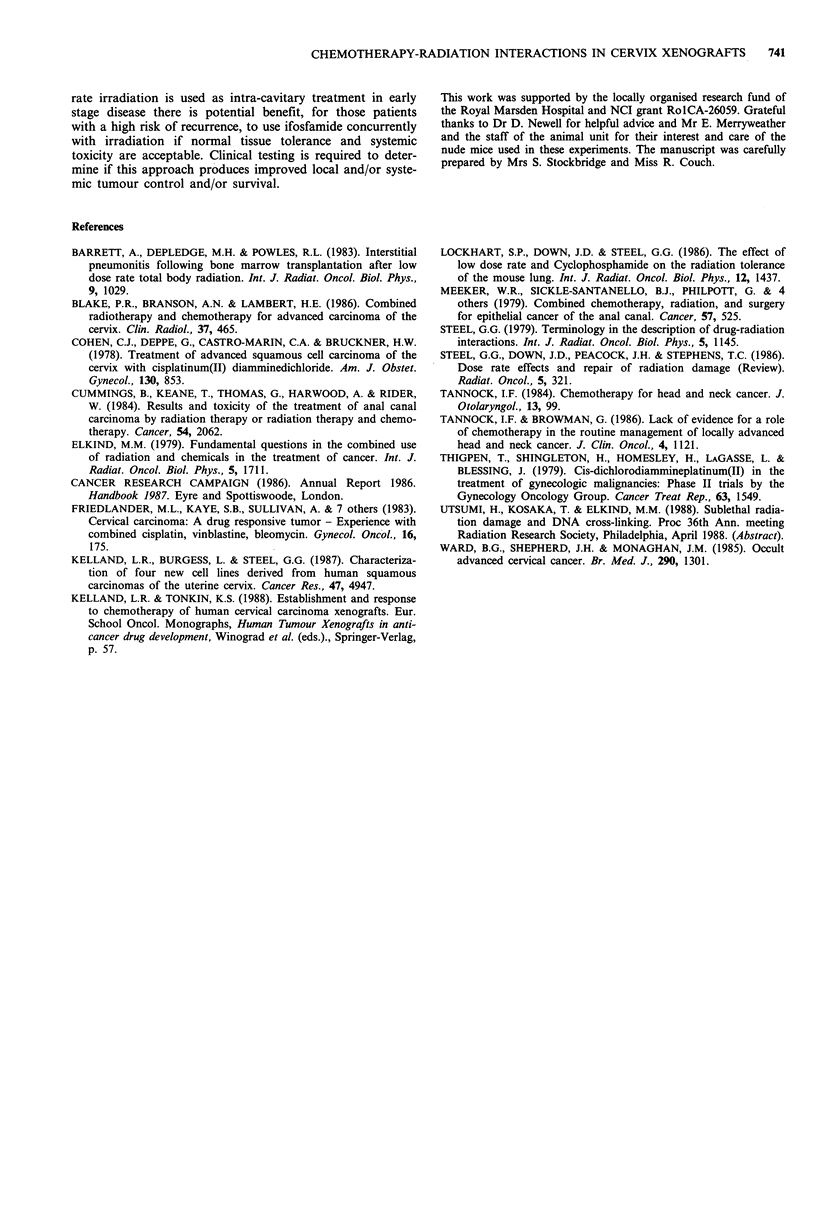# Chemotherapy-radiation interactions in human cervix carcinoma xenografts.

**DOI:** 10.1038/bjc.1988.300

**Published:** 1988-12

**Authors:** K. S. Tonkin, L. R. Kelland, G. G. Steel

**Affiliations:** Radiotherapy Research Unit, Institute of Cancer Research, Sutton, Surrey, UK.

## Abstract

The combination of irradiation and four agents of clinical interest in the treatment of cervix carcinoma (bleomycin, etoposide, cisplatin and ifosfamide) have been investigated using two human cervix carcinoma xenografts in nude mice. As a model of clinical brachytherapy regimes, radiation was administered at a continuous low dose rate of 5 cGy min-1 to a total dose of 9 or 12 Gy. No substantial enhancement in tumour growth delay over that observed for radiation alone was observed with bleomycin, etoposide or cisplatin. Ifosfamide, however, led to substantial additional growth delay, an effect which was lost when irradiation was administered at a higher dose rate of 70 cGy min-1. As dose-rates of around 5 cGy min-1 allow greater repair of radiation damage than at the higher dose-rate without significant cell cycling or repopulation, it is possible that ifosfamide may act as an inhibitor of repair processes in this model. It would be of interest to evaluate the role of ifosfamide and brachytherapy regimes in the clinical treatment of carcinoma of the cervix.


*Br. J. Cancer* (1988), **58**, 738–741 © The Macmillan Press Ltd., 1988

### Chemotherapy-radiation interactions in human cervix carcinoma xenografts

K.S. Tonkin*, L.R. Kelland & G.G. Steel

*Radiotherapy Research Unit, Institute of Cancer Research, Cotswold Road, Sutton, Surrey SM2 5NG, UK.*

**Summary** The combination of irradiation and four agents of clinical interest in the treatment of cervix carcinoma (bleomycin, etoposide, cisplatin and ifosfamide) have been investigated using two human cervix carcinoma xenografts in nude mice. As a model of clinical brachytherapy regimes, radiation was administered at a continuous low dose rate of $5\,cGy\,min^{-1}$ to a total dose of 9 or 12 Gy. No substantial enhancement in tumour growth delay over that observed for radiation alone was observed with bleomycin, etoposide or cisplatin. Ifosfamide, however, led to substantial additional growth delay, an effect which was lost when irradiation was administered at a higher dose rate of $70\,cGy\,min^{-1}$. As dose-rates of around $5\,cGy\,min^{-1}$ allow greater repair of radiation damage than at the higher dose-rate without significant cell cycling or repopulation, it is possible that ifosfamide may act as an inhibitor of repair processes in this model. It would be of interest to evaluate the role of ifosfamide and brachytherapy regimes in the clinical treatment of carcinoma of the cervix.

Carcinoma of the uterine cervix is the third most frequently occurring female gynaecological malignancy. Overall it accounts for 6% of female malignancies. In England and Wales the incidence is 4,400 new cases per year with an overall survival of 57% (Cancer Research Campaign, 1986). With 160,000 deaths per year from all cancers this means that carcinoma of the cervix accounts for 3% of cancer deaths.

Although chemotherapy for metastatic disease does not achieve long-term survival the response rates are relatively high. If chemotherapy were used in a more favourable situation one might hope to improve outcome (Ward *et al.*, 1985). Thus chemotherapy is being tested in the adjuvant setting along with either surgery or radiotherapy where one of the problems of access of chemotherapy to post-irradiated tissue is eliminated.

The present paper describes the interaction between commonly used chemotherapeutic agents (bleomycin, cisplatin, etoposide and ifosfamide) and low dose-rate irradiation ($5\,cGy\,min^{-1}$) in two recently established carcinoma of the cervix cell lines used in xenograft. These drugs have been used extensively in clinical trials both singly and in combination (Blake *et al.*, 1986; Cohen *et al.*, 1978; Thigpen *et al.* 1979; Friedlander *et al.*, 1983). We have chosen to use low dose-rate irradiation at $5\,cGy\,min^{-1}$ on the basis of *in vitro* studies from this laboratory that have shown doses of 2-$5\,cGy\,min^{-1}$ allow extensive recovery from radiation damage without significant repopulation or cell cycle progression (Steel *et al.*, 1986). In the clinical setting brachytherapy to the cervix is given at approximately 10 Gy to 30 Gy/24 h which is $0.7\,cGy\,min^{-1}$ to $2\,cGy\,min^{-1}$. In these experiments the low dose-rate of $5\,cGy\,min^{-1}$ enabled the exposure time to be kept below four hours (total dose 12 Gy), thus minimising stress to the mice.

The aim was to evaluate chemotherapy induced improvement in tumour growth delay in this experimental system while monitoring any increased toxicity of such combined modality treatment using body weight measurements and mortality (Tannock, 1984, 1986).

### Materials and methods

### *Xenografts*

Two recently established carcinoma of the cervix cell lines HX155c and HX156c were used. The biological characteristics of these cell lines and the xenograft lines, which were established from initial pre-treatment biopsies taken from patients presenting to The Royal Marsden Hospital, have been described previously (Kelland *et al.*, 1987, for the cell lines; and Kelland & Tonkin, 1988, for the xenograft lines). The tumour lines were passaged in female Nu/Nu mice which were housed in negatively pressured plastic film isolators and fed on standard animal chow and water. Animals were removed from the isolator and put in a sterile hood for transplantation. The mice were placed under ether anaesthesia and ear-marked at this time. An incision was made over the lumbar spine area and a 1–2 mm tumour nodule was implanted subcutaneously over the dorsal spine. In order to keep the tumour in position a skin tuck was made with a skin clip just posterior to the nodule and a second clip was used to close the wound. The mice were observed for 4 to 12 weeks when the clips were removed from suitably positioned tumours of 6–9 mm in diameter.

### *Chemotherapeutic agents*

We used maximum tolerated doses of drug for nude mice determined previously in the department or evaluated immediately prior to these experiments. All drugs were dissolved and dilutions made in PBS with 0.6 ml as the maximum volume of injection. All control mice were given PBS as a single i.p. injection.

Etoposide vehicle was made up as a $2\,mg\,ml^{-1}$ solution of polyethyleneglycol, tween 80, citric acid and absolute alcohol. Cisplatin, etoposide and ifosfamide were all given within 2 h of the start of irradiation. Bleomycin was administered 2 days prior to irradiation as preliminary 'time-line' experiments showed this to be optimal for achieving maximum antitumour activity when combined with radiation. Experiments were done using a control, drug only, irradiation only and drug with irradiation arms where the control and irradiation alone groups received a single i.p. injection of PBS at the same time as the drug was given to the other mice.

Drug doses were: bleomycin $150\,\mu g\,g^{-1}$; cisplatin $5\,\mu g\,g^{-1}$; Etoposide $60\,\mu g\,g^{-1}$; ifosfamide $120\,\mu g\,g^{-1}$.

### *Irradiation*

For irradiation each mouse was put in an individual perspex holder and placed behind lead shielding to protect normal tissues. Low dose-rate experiments were performed at $5\,cGy\,min^{-1}$ using a $^{60}Co$ $\gamma$-radiation source (100 Ci). The mice were positioned in a line of 5 per dose-rate tested. The

Correspondence: L.R. Kelland.
*Present address: Department of Oncology, Charing Cross Hospital, Fulham Palace Road, London SW6, UK.
Received 23 April 1988; and in revised form, 26 July 1988.

maximum duration of experiment for low dose-rate irradiation was 4 h. A total dose of 9 Gy irradiation at 5 cGy min$^{-1}$ was delivered from the 100 Ci $^{60}$Co source for all experiments except with etoposide where a total dose of 12 Gy was used at the same dose-rate. High dose-rate experiments were done at 70 cGy min$^{-1}$ using a large $^{60}$Co $\gamma$-radiation source (2000 Ci) and all these experiments were complete in <20 min.

*Experimental endpoint and statistical analysis*

Tumours were measured in two perpendicular diameters using calipers and tumour weight was calculated from a calibration curve. The weight of each mouse was documented at each measurement. The results are shown as Relative Tumour Weight on a logarithmic scale plotted against time in days after the day of irradiation. The graphs represent mean values for 5 to 15 individual tumours and the error bars for the Ifosfamide experiments indicate the standard error of the mean (s.e.). The Specific Growth Delay (SGD) values were calculated from

$$\text{Specific Growth Delay (SGD)} = \frac{T_2 - T_1}{D_t}$$

where $T_2$ is the time to reach twice tumour weight in the treated tumours in days and $T_1$ is the time in days to reach twice tumour weight in the control tumours. $D_t$ the doubling time of the control tumours in days.

### Results

The results for HX155 with bleomycin and cisplatin are shown in Figures 1a and 1b and Table I. Cisplatin alone (Figure 1b) appeared to cause little tumour growth delay. However, bleomycin alone (Figure 1a) resulted in a specific growth delay (SGD) of 1.3. Irradiation alone gave a SGD of 3 to 3.5 in both experiments but the addition of either drug did not appear to result in a significantly greater SGD than that observed with radiation alone.

The results of HX156 with bleomycin and etoposide are shown in Figure 1c, d respectively and Table I. There was a maximum SGD of 1.3 for bleomycin and irradiation but this was not significantly different from the SGD for bleomycin or irradiation alone and much less than the SGD noted for HX155. As a consequence of these results the etoposide experiments using HX156 (Figure 1d) were performed with a total dose of 12 Gy radiation rather than 9 Gy but there was little obvious improvement in SGD when drug was combined with irradiation than for irradiation alone.

In the experiments with low dose-rate irradiation and ifosfamide there was substantial additional growth delay with combined treatment (Figure 2a; Table II). The SGD for irradiation was 2.3 and for combined irradiation and ifosfamide the SGD was 10. There was only little tumour growth delay for ifosfamide alone.

To further evaluate the effect of combined modality treatment involving ifosfamide, the experiments were repeated using 9 Gy total dose irradiation given at a high dose-rate of 70 cGy min$^{-1}$ on a 2000 Ci $^{60}$Co source. The result is shown in Figure 2b and Table II and demonstrated that high dose-rate irradiation with ifosfamide gave similar SGD as irradiation alone with values of 4-4.3. Toxicity monitoring revealed no additional weight loss in mice when combined treatment was given than with chemotherapy or irradiation alone and no treatment related deaths were observed.

### Discussion

In combining radiotherapy and chemotherapy one aims to improve the response rate achieved by either modality alone.

**Figure 1** Tumour growth delay for xenograft tumours in nude mice with chemotherapy and/or irradiation.

1a HX155; 1b HX155; 1c HX156; 1d HX156.

The difficulty is to achieve a higher response without unacceptable toxicity, that is, to improve the therapeutic ratio. Four mechanisms have been used to describe the ways in which an improved therapeutic ratio could be achieved. They are, spatial co-operation, independent cell kill, normal tissue protection and enhancement of tumour response (Steel, 1979).

In the experiments reported here several commonly used chemotherapeutic agents have been used with low dose-rate irradiation (etoposide, cisplatin, bleomycin and ifosfamide). Only ifosfamide and irradiation resulted in a much greater growth delay than irradiation alone. These experiments demonstrated an additive response of low dose-rate irradiation and ifosfamide giving a SGD of 10 with a SGD of 2.3 for irradiation alone in comparison to the high dose-rate

**Table I** Specific Growth Delay (SGD) for chemotherapy and low dose-rate irradiation

| *Cell line designation* | *Control tumour doubling time (days)* | *Irradiation*[a] | *Treatment chemotherapy* | *Irradiation and chemotherapy* |
|---|---|---|---|---|
| | | | Bleomycin (150 $\mu g\,g^{-1}$) | |
| HX155 | 8 | 3.0 | 1.3 | 3.5 |
| HX156 | 6 | 0.7 | 0.7 | 1.3 |
| | | | Cisplatin (5 $\mu g\,g^{-1}$) | |
| HX155 | 6.5 | 2.3 | 0.3 | 2.7 |
| | | | Etoposide (60 $\mu g\,g^{-1}$) | |
| HX156 | 15 | 1.3 | 0.3 | 1.3 |

[a]Irradiation was 9 Gy total dose except HX156, etoposide when 12 Gy total dose was administered.

**Figure 2** Tumour growth delay of HX155 xenograft tumours in nude mice with Ifosfamide and irradiation. Low dose-rate (5 cGy min$^{-1}$) irradiation: (a) and high dose-rate (70 cGy min$^{-1}$) irradiation; (b) Control (●) Ifosfamide (○) Irradiation (■) Ifosfamide and irradiation (□).

irradiation experiments which resulted in an SGD of 4-4.3 for both irradiation and irradiation and ifosfamide.

Using the terminology of Steel this result is called 'enhancement'. This describes a positive interaction where the dose response curve for only one modality is known. In this instance only the dose response for irradiation has been established in the nude mouse. The result of combined treatment was marked tumour growth delay where either modality alone did not achieve a substantial effect. When ifosfamide was given with high dose-rate irradiation the tumour growth delay achieved by the combination of drug

**Table II** Specific Growth Delay (SGD) for ifosfamide and irradiation in HX155

| *Treatment* | *Specific growth delay* |
|---|---|
| Ifosfamide (120 $\mu g\,g^{-1}$) | 0.9 |
| Low dose-rate irradiation (5 cGy min$^{-1}$ 9 Gy total dose) | 2.3 |
| High dose-rate irradiation (70 cGy min$^{-1}$ 9 Gy total dose) | 4.0 |
| Ifosfamide and low dose-rate irradiation | 10.0 |
| Ifosfamide and high dose-rate irradiation | 4.3 |

Mean control (untreated) tumour doubling times were 7 days for the low dose-rate experiments and 9 days for the high dose-rate experiments.

and irradiation was no different to that achieved by irradiation alone. In addition the growth delay for high dose-rate irradiation, with or without Ifosfamide, was less than the growth delay for the combination of low-dose rate irradiation and drug by a factor of four. This suggests that the mechanism by which ifosfamide acts with low dose-rate irradiation, might involve inhibition of the repair that would normally be seen with low dose-rate irradiation given alone. The concept of the loss of low dose-rate sparing has been reported from this laboratory previously when lung tolerance was investigated using irradiation and cyclophosphamide (Lockhart *et al.*, 1986) and the potential for lung damage when irradiation is combined with cyclophosphamide in bone marrow transplantation is well documented (Barrett *et al.*, 1983).

As far as we are aware this is the first report of increased tumour growth delay with chemotherapy and low dose-rate irradiation in a human tumour model. More data, particularly *in vitro* using tumour cell lines, are needed to evaluate the mechanism of this interaction. Ifosfamide is an alkylating agent which causes cell death largely as a result of its ability to form cross-links in DNA, although it also acts by substitution reactions, base alkylation and phosphate group esterification. Radiation is known to cause base damage, single and double stranded DNA breaks and DNA-DNA and DNA-protein cross-links (Elkind, 1979 for a review). In addition there is some recent evidence (Utsumi *et al.*, 1988) to suggest that, in S-phase cells, radiation-induced sublethal damage may be a cross-linking lesion. It is possible that, during continuous low dose-rate irradiation, ifosfamide could be inhibiting the repair of such a lesion. Further study is required to elucidate the mechanism for the striking enhancement in observed tumour growth delay.

To date there has been a paucity of data to support the use of radiotherapy and chemotherapy concurrently in solid tumours as patient survival is rarely improved and greater toxicity usually results (Tannock, 1984; 1986). However in some squamous cell tumours such as carcinoma of the anal canal there have been encouraging reports of improved local control with combined treatment using 5-Fluorouracil (5-Fu) (Cummings *et al.*, 1984; Meeker *et al.*, 1986).

In patients with carcinoma of the cervix where low doserate irradiation is used as intra-cavitary treatment in early stage disease there is potential benefit, for those patients with a high risk of recurrence, to use ifosfamide concurrently with irradiation if normal tissue tolerance and systemic toxicity are acceptable. Clinical testing is required to determine if this approach produces improved local and/or systemic tumour control and/or survival.

This work was supported by the locally organised research fund of the Royal Marsden Hospital and NCI grant Ro1CA-26059. Grateful thanks to Dr D. Newell for helpful advice and Mr E. Merryweather and the staff of the animal unit for their interest and care of the nude mice used in these experiments. The manuscript was carefully prepared by Mrs S. Stockbridge and Miss R. Couch.

### References

BARRETT, A., DEPLEDGE, M.H. & POWLES, R.L. (1983). Interstitial pneumonitis following bone marrow transplantation after low dose rate total body radiation. *Int. J. Radiat. Oncol. Biol. Phys.*, **9**, 1029.

BLAKE, P.R., BRANSON, A.N. & LAMBERT, H.E. (1986). Combined radiotherapy and chemotherapy for advanced carcinoma of the cervix. *Clin. Radiol.*, **37**, 465.

COHEN, C.J., DEPPE, G., CASTRO-MARIN, C.A. & BRUCKNER, H.W. (1978). Treatment of advanced squamous cell carcinoma of the cervix with cisplatinum(II) diamminedichloride. *Am. J. Obstet. Gynecol.*, **130**, 853.

CUMMINGS, B., KEANE, T., THOMAS, G., HARWOOD, A. & RIDER, W. (1984). Results and toxicity of the treatment of anal canal carcinoma by radiation therapy or radiation therapy and chemotherapy. *Cancer*, **54**, 2062.

ELKIND, M.M. (1979). Fundamental questions in the combined use of radiation and chemicals in the treatment of cancer. *Int. J. Radiat. Oncol. Biol. Phys.*, **5**, 1711.

CANCER RESEARCH CAMPAIGN (1986). Annual Report 1986. *Handbook 1987*. Eyre and Spottiswoode, London.

FRIEDLANDER, M.L., KAYE, S.B., SULLIVAN, A. & 7 others (1983). Cervical carcinoma: A drug responsive tumor – Experience with combined cisplatin, vinblastine, bleomycin. *Gynecol. Oncol.*, **16**, 175.

KELLAND, L.R., BURGESS, L. & STEEL, G.G. (1987). Characterization of four new cell lines derived from human squamous carcinomas of the uterine cervix. *Cancer Res.*, **47**, 4947.

KELLAND, L.R. & TONKIN, K.S. (1988). Establishment and response to chemotherapy of human cervical carcinoma xenografts. Eur. School Oncol. Monographs, *Human Tumour Xenografts in anticancer drug development*, Winograd *et al.* (eds.), Springer-Verlag, p. 57.

LOCKHART, S.P., DOWN, J.D. & STEEL, G.G. (1986). The effect of low dose rate and Cyclophosphamide on the radiation tolerance of the mouse lung. *Int. J. Radiat. Oncol. Biol. Phys.*, **12**, 1437.

MEEKER, W.R., SICKLE-SANTANELLO, B.J., PHILPOTT, G. & 4 others (1979). Combined chemotherapy, radiation, and surgery for epithelial cancer of the anal canal. *Cancer*, **57**, 525.

STEEL, G.G. (1979). Terminology in the description of drug-radiation interactions. *Int. J. Radiat. Oncol. Biol. Phys.*, **5**, 1145.

STEEL, G.G., DOWN, J.D., PEACOCK, J.H. & STEPHENS, T.C. (1986). Dose rate effects and repair of radiation damage (Review). *Radiat. Oncol.*, **5**, 321.

TANNOCK, I.F. (1984). Chemotherapy for head and neck cancer. *J. Otolaryngol.*, **13**, 99.

TANNOCK, I.F. & BROWMAN, G. (1986). Lack of evidence for a role of chemotherapy in the routine management of locally advanced head and neck cancer. *J. Clin. Oncol.*, **4**, 1121.

THIGPEN, T., SHINGLETON, H., HOMESLEY, H., LAGASSE, L. & BLESSING, J. (1979). Cis-dichlorodiammineplatinum(II) in the treatment of gynecologic malignancies: Phase II trials by the Gynecology Oncology Group. *Cancer Treat Rep.*, **63**, 1549.

UTSUMI, H., KOSAKA, T. & ELKIND, M.M. (1988). Sublethal radiation damage and DNA cross-linking. Proc 36th Ann. meeting Radiation Research Society, Philadelphia, April 1988. (*Abstract*).

WARD, B.G., SHEPHERD, J.H. & MONAGHAN, J.M. (1985). Occult advanced cervical cancer. *Br. Med. J.*, **290**, 1301.